# Murine *Cep290* phenotypes are modified by genetic backgrounds and provide an impetus for investigating disease modifier alleles

**DOI:** 10.12688/f1000research.6959.1

**Published:** 2015-08-20

**Authors:** Simon Ramsbottom, Colin Miles, John Sayer

**Affiliations:** 1 Institute of Genetic Medicine, Newcastle University, Newcastle upon Tyne, NE1 3BZ, UK

**Keywords:** Joubert syndrome, Cep290, cilia, modifier, cystic kidney, nephronophthisis

## Abstract

The study of primary cilia is of broad interest both in terms of disease pathogenesis and the fundamental biological role of these structures. Murine models of ciliopathies provide valuable tools for the study of these diseases. However, it is important to consider the precise phenotype of murine models and how dependant it is upon genetic background. Here we compare and contrast murine models of *Cep290*, a frequent genetic cause of Joubert syndrome in order to refine our concept of genotype-phenotype correlations.

## Background

The role of cilia and the diseases associated with aberrant cilial formation and function, termed ciliopathies, have in the last ten years become a major area of study ^1^. Within the field of human genetics, the understanding of the importance of these organelles has given rise to a profound shift in the way that the study and treatment of a range of diseases is undertaken. Many syndromes, although known to be pleiotropic in their manifestation, were previously considered to be discrete entities requiring specific individualised treatment. The discovery that many of these syndromes appear to have a large degree of commonality in their disease mechanism via the malformation or mis-localisation of cilia, has lead to the thinking that they may indeed exist within a spectrum, and subsequently that they may respond to similar treatments.

Joubert syndrome (JBTS) is an autosomal recessive ciliopathy which gives rise to cerebellar vermis aplasia, hypotonia, ataxia and developmental delay. It is often associated with retinal degeneration, leading to blindness. It may also be associated with a cystic renal phenotype known as nephronophthisis (NPHP). The most common genetic lesion among patients with JBTS who present with the cerebello-oculo-renal phenotype is *CEP290* (OMIM 610142) ^2^. Mutations in the *CEP290* gene are associated with numerous disorders including Leber congenital amaurosis ^3^, Senior-Loken syndrome, JBTS ^4, 5^ and Meckel syndrome ^6^. There have been over 100 different mutation sites reported in human patients ^7^. Work attempting to understand genotype-phenotype correlations of *CEP290* mutations based on total amount of protein has recently been reported ^8^. A lack of correlation between genotype and phenotype may also be dependent on oligogenic inheritance and a mutational load in ciliary genes ^9^. While this diversity has been suggested to contribute to these differential outcomes, recent work utilising mouse models of JBTS has indicated that there may be underlying mechanisms that modify the disease phenotype, even when the exact *Cep290* mutation site is conserved.

In early 2015, Rachel *et al*. described a *Cep290 ^KO^* mouse model of JBTS, which exhibits retinal degeneration and hydrocephalus in juvenile mice, with a slowly progressive renal pathology resulting in cysts in adult mice ^10^. While the retinal degeneration and cerebral phenotypes broadly reflect those observed in JBTS patients, the murine kidney phenotype is unusual, as in humans it typically presents in teenage years and young adults as opposed to being of late onset. This lack of a significant renal aspect in the pathology may simply reflect the diversity in the JBTS phenotype. It should be noted however that while a renal phenotype in JBTS is not universal, patients with mutations in *CEP290*, as opposed to other JBTS genes, do more commonly have renal disease (nephronophthisis) ^11^.

Rachel *et al.* report that a significant number (80%) of *Cep290 ^KO^* mice do not survive past weaning, suggesting that the reported phenotype is that of less affected individuals. Additionally, to obtain viable mice, the authors propagated the mutation within a mixed background of C57BL/6 and 129/SvJ; mice could not be bred purely on either line past the N3 generation. While this genetic diversity in the population may echo that of a human scenario, it can be difficult to interpret data with the lack of experimental control that this diversity introduces. Furthermore it is likely that to fully ascertain an accurate picture of the disease model, it is necessary to use a significantly greater number of animals, which is not desirable. To investigate a wider phenotypic spectrum of Cep290-related disorders, Rachel *et al.* also use a gene trap (gt) model of *Cep290*, in which the pGT0xfT2 gene trap vector is inserted in intron 25, resulting in the introduction of a premature stop codon ^10^. This lesion is similar to that of a number of common mutation sites within human *CEP290*, and so potentially provides a good model of the human disease. *Cep290 ^gt^* were backcrossed onto 129SvJ and Rachel *et al.* reported a significant level of lethality, as was reported for the *Cep290 ^KO^* model, with the vast majority of *Cep290 ^gt^* mice dying *in utero* between E12 and E14. Surviving mice displayed massively dilated kidneys, with loss of tubules and the formation of large cysts, a phenotype more similar to that of infantile nephronophthisis. As with the *Cep290 ^KO^* model however, the large mortality rate within the population needs to be considered, as those mice that do survive may not be fully representative.

This gene trap model ( *Cep290 ^gt^*) is similar to a murine model reported in 2014, described as *Cep290 ^LacZ^* ^12^. In this study, a similarly high level of *in utero* mortality was reported when *Cep290 ^LacZ^* mice were backcrossed on to a C57BL/6 background. On the 129/Ola background however mice were fertile and viable beyond 12 months ^12^. Although the two studies generated mice from the same embryonic gene trap cell line (CC0582 [SIGTR, http://www.sanger.ac.uk/resources/mouse/sigtr/]), differences in the phenotype can be observed. While the retinal degeneration and ventriculomegaly were broadly similar between the two studies, kidneys of *Cep290 ^LacZ^* mice were not massively dilated as described by Rachel *et al.*, but had progressive formation of cysts reminiscent of human nephronophthisis ^12^. Cysts were observed in newborn mice, and became progressively larger over the first 4 weeks, instead of developing after 12 months. This disparity in the viability and phenotype of mice containing the same genetic alteration indicates that there are significant genetic modifiers specific to each strain of mice which can adversely affect the way in which this disease presents. The genetic diversity present between human populations therefore may provide some answers as to the broad range of phenotypes seen in JBTS patients. In the case of mutations such as those in *Cep290* which do have pleiotropic effects, the difference between the outcomes in different mouse strains provides an opportunity to study how genetic variability affects the disease phenotype. By utilising strains with known discrete polymorphisms it should be possible to identify variants that correlate with specific outcomes, allowing for a much greater understanding of the role that specific genetic modifiers play in human disease and allowing therapies to be directed at these genetic modifiers.
