# Supplementary material for: Murine Cep290 phenotypes are modified by genetic backgrounds and provide an impetus for investigating disease modifier alleles
Source: F1000Res. 2015 Aug 20;4:590. [Version 1] doi: 10.12688/f1000research.6959.1 (PMC4648220; doi:10.12688/f1000research.6959.1)
Supplement: Peer review history

# Peer review: Referee response for version 1

**DOI:** 10.5256/f1000research.7495.r10185

**Published:** 2015-09-22

**Authors:** Russell J. Ferland, Kiet Hua

**Affiliations:** 1 Center for Neuropharmacology and Neuroscience and the Department of Neurology, Albany Medical College, Albany, NY, USA; 2 Center for Neuropharmacology and Neuroscience, Albany Medical College, Albany, NY, USA

In recent years, it has become increasingly more evident that many ciliopathies exist not as separate diseases, but instead fall along a spectrum. *Meckel syndrome 1 (MKS1)* mutations may cause the severe Meckel Gruber syndrome, but recently, has been reported by Romani *et al.* (2014) to also be responsible for cases of the relatively more mild Joubert syndrome (JBTS). Our research on the *centrosome and spindle pole associated protein 1 (CSPP1)* gene supports the notion that mutations within the same gene can carry a wide range of phenotypes (Tuz *et al.*, 2015).  We identified 18 individuals with JBTS from 14 families with biallelic truncating mutations in *CSPP1*, and saw no evidence of a correlation between phenotype and the site of truncation. Surprisingly, individuals with the most N-terminal truncations displayed the least severe phenotype. Our findings also revealed that siblings with the same mutations had varying phenotypes, suggesting that other factors outside the mutation of the ciliopathy gene itself plays a role in the ultimate phenotype of the affected individual.

The phenotypic heterogeneity of individuals with JBTS with the same mutations has not gone unnoticed. Recently, Ben-Omran *et al*. (2015) described two Qatari families with histories of consanguineous marriages with the same *CSPP1* mutation, but yet, the probands displayed clinical heterogeneity. Ben-Omran and colleagues (2015) argue that highly inbred families likely have less diversity in genetic modifiers, and attribute their findings of clinical heterogeneity to unidentified stochastic events that occur in development. They correctly acknowledge that a disadvantage of their study is the lack of identical twins.

Murine ciliopathy models provide one way of controlling for the contribution of genetic background to the eventual phenotype of the affected individual. In this correspondence from Ramsbottom, Miles, and Sayer, the authors provide important insight into the role of genetic background in modifying transgenic ciliopathy phenotypes. Here, the authors reflect on a recent publication in the journal Human Molecular Genetics by Rachel *et al. *(2015) which describes different ciliopathy phenotypes in mice with different *Cep290 *mutations (a constitutive deletion of Cep290 (proposed null) and a genetrap allele (proposed truncated Cep290)). Moreover, the phenotypes were clearly modified by the genetic background of the mice. Further support for this came from comparisons of these phenotypes to other published *Cep290 *transgenic mice with different genetic alterations. Genetic background and the modifiers that can be in these backgrounds is a significant consideration that needs to be appreciated in the cilia field as it has in many other fields, such as behavioral neuroscience. In fact, we have published work in which we have shown that C57BL/6J mice with a targeted deletion of *Ahi1*, another gene causing Joubert syndrome, die within 12 hours after birth, but if these mice are backcrossed to FVB, Balbc/J, or Swiss Webster mice (>10 generations), then post-natal survivability is significantly enhanced and most mice can reach adulthood. This correspondence serves as a very important reminder that the genetic background on which you are studying your ciliopathy phenotype can result in different phenotypes dependent on the mouse strain and therefore needs to be considered in interpreting one's results. Consequently, identification of such differing ciliopathy phenotypes on different genetic backgrounds also serve as an important tool for not only potentially identifying modifiers of ciliopathy phenotypes, but may also result in the identification of new ciliopathy genes.

Ramsbottom, Miles, and Sayer argue that a difference in genetic background could account for disparities in some phenotypes amongst individuals with the same mutation, a finding that is supported by our work in humans and mice. However, as the authors point out, murine models of ciliopathies do not always recapitulate the findings in affected individuals with JBTS. Thus, a possible difference between murine and human regulation of ciliopathy phenotype must also be considered.

We have read this submission. We believe that we have an appropriate level of expertise to confirm that it is of an acceptable scientific standard.

# Peer review: Referee response for version 1

**DOI:** 10.5256/f1000research.7495.r10029

**Published:** 2015-08-25

**Authors:** Eugen Boltshauser

**Affiliations:** 1 Department of Pediatric Neurology, University Children's Hospital Zurich, Zurich, Switzerland

In this correspondence Ramsbottom and colleagues refer to a publication by Rachel *et al. *(2015) in which phenotypes in mice strains with mutations in Cep290 (knock-out and a gen trap model) were studied, and a comparison with patients with Joubert syndrome (JBTS) with CEP290 mutations is made. In brief: mice develop a retinal degeneration, hydrocephalus, and a rather late onset progressive renal pathology, while JBTS patients develop kidney disease in childhood or early adulthood. In addition the majority of knock-out mice do not survive past weaning, thus the survivors are probably not really representative of the phenotype, Ramsbottom *et al *argue that differences between the outcomes in different mouse strains provide an opportunity to study how genetic variability affects the disease phenotype. This point is well taken. Looking at affected children (in my professional activity) even within the same sibship, with identical mutations, there is often marked intrafamilial variability of the phenotype, pointing to the relevance of genetic modifiers and probable interaction with other cilia-genes. Studies of mouse strains (and zebra fish) have greatly contributed to understanding Joubert related genes, however the animal phenotype may differ from humans - as illustrated by the lack of hydrocephalus in CEP290 mutated JBTS patients.

I have read this submission. I believe that I have an appropriate level of expertise to confirm that it is of an acceptable scientific standard.
